# Ovulatory signals alter granulosa cell behavior through YAP1 signaling

**DOI:** 10.1186/s12958-019-0552-1

**Published:** 2019-12-28

**Authors:** Tianyanxin Sun, Francisco J. Diaz

**Affiliations:** 1 Department of Obstetrics and Gynecology, Stanford University School of Medicine, Stanford, CA 94305 USA; 2 Center for Reproductive Biology and Health and Department of Animal Science, The Pennsylvania State University, 313 FRL Building, University Park, PA 16802 USA

**Keywords:** Ovulation, Granulosa cells, COCs, Hippo, YAP1, Verteporfin, Cell survival, Differentiation

## Abstract

**Background:**

The Hippo pathway plays critical roles in regulating cell proliferation, differentiation and survival among species. Hippo pathway proteins are expressed in the ovary and are involved in ovarian function. Deletion of *Lats1* causes germ cell loss, ovarian stromal tumors and reduced fertility. Ovarian fragmentation induces nuclear YAP1 accumulation and increased follicular development. At ovulation, follicular cells stop proliferating and terminally differentiate, but the mechanisms controlling this transition are not completely known. Here we explore the role of Hippo signaling in mouse granulosa cells before and during ovulation.

**Methods:**

To assess the effect of oocytes on Hippo transcripts in cumulus cells, cumulus granulosa cells were cultured with oocytes and cumulus oocyte complexes (COCs) were cultured with a pSMAD2/3 inhibitor. Secondly, to evaluate the criticality of YAP1 on granulosa cell proliferation, mural granulosa cells were cultured with oocytes, YAP1-TEAD inhibitor verteporfin or both, followed by cell viability assay. Next, COCs were cultured with verteporfin to reveal its role during cumulus expansion. Media progesterone levels were measured using ELISA assay and Hippo transcripts and expansion signatures from COCs were assessed. Lastly, the effects of ovulatory signals (EGF in vitro and hCG in vivo) on Hippo protein levels and phosphorylation were examined. Throughout, transcripts were quantified by qRT-PCR and proteins were quantified by immunoblotting. Data were analyzed by student’s t-test or one-way ANOVA followed by Tukey’s post-hoc test or Dunnett’s post-hoc test.

**Results:**

Our data show that before ovulation oocytes inhibit expression of Hippo transcripts and promote granulosa cell survival likely through YAP1. Moreover, the YAP1 inhibitor verteporfin, triggers premature differentiation as indicated by upregulation of expansion transcripts and increased progesterone production from COCs in vitro. In vivo, ovulatory signals cause an increase in abundance of Hippo transcripts and stimulate Hippo pathway activity as indicated by increased phosphorylation of the Hippo targets YAP1 and WWTR1 in the ovary. In vitro, EGF causes a transient increase in YAP1 phosphorylation followed by decreased YAP1 protein with only modest effects on WWTR1 in COCs.

**Conclusions:**

Our results support a YAP1-mediated mechanism that controls cell survival and differentiation of granulosa cells during ovulation.

## Background

The cumulus-oocyte complex (COC) and the ovarian follicle undergo dramatic transformations during the peri-ovulatory transition. In the absence of ovulatory signals, the oocyte is maintained in meiotic arrest while the granulosa cells are highly proliferative but susceptible to apoptosis. Ovulatory signals reverse this process and cause granulosa cells to stop proliferating and differentiate into luteal cells or expanded cumulus cells both of which produce progesterone [1]. Oocyte secreted factors define the cumulus cell phenotype by promoting proliferation [2, 3] and survival [4], while suppressing luteinization [5–8]. Many of the effects of oocytes on cumulus cells are mediated, in part, by oocyte-specific factors, such as GDF9 and BMP15, which activate the SMAD2/3 and SMAD1/5/9 signaling pathways [7, 9–12]. Cumulus cells, in turn, promote developmental competence of the oocyte [13–24]. The cumulus cell-derived factors that promote oocyte development are less well-understood, but include factors that regulate transcriptional silencing [25], meiotic arrest [26, 27] and free intracellular zinc [28].

The LH surge initiates a signaling cascade that profoundly transforms the cellular phenotype of both mural and cumulus granulosa cells. One important pathway activated by the LH surge is activation of MAPK signaling pathway, mediated through an increase in Epidermal Growth Factor (EGF)-like peptides [1, 29–31]. In the cumulus cells, activation of the EGF signaling pathway initiates the process of cumulus expansion [7], causes cells to exit from the cell cycle and increases resistance to apoptosis [32–35]. In addition, EGF causes the up-regulation of progesterone from the cumulus cells [1] which may serve as a sperm chemoattractant factor during fertilization and/or may be important for oocyte nuclear maturation [36–39]. Thus, granulosa cells transition from highly proliferative and un-differentiated phenotype into terminally differentiated cells with little capacity to proliferate. These dramatic transformations of the somatic follicular cells in the pre-ovulatory follicle are critical for optimal fertility and ovarian function, but the downstream intra-follicular mechanisms mediating these responses are not completely known.

The Hippo pathway is a key regulator of the cell fate decision to proliferate, remain quiescent or undergo cell death [40]. Activation of the Hippo kinases, STK3 and STK4 (MST1/2) leads to a kinase cascade including phosphorylation and activation of the kinases LATS1 and LATS2, which in turn, phosphorylates and inactivates the transcriptional co-activators, Yes associated protein (YAP1) and WWTR1 (also known as TAZ). Phosphorylated YAP1 and WWTR1 proteins are sequestered in the cytoplasm and are prevented from acting as transcriptional co-activators. When the Hippo pathway is suppressed, unphosphorylated YAP1 and WWTR1 move into the nucleus where they activate genes involved in survival and proliferation [41, 42]. Given its role in proliferation, it is not surprising that the Hippo pathway is important for the regulation and maintenance of various stem cell populations [43, 44]. Early studies found that disruption of various Hippo pathway components caused increased organ size in drosophila [45]. In mammals, liver-specific deletion of *Stk4*^*−/−*^*Stk3*^*−/−*^ causes up-regulation of YAP1 and increases liver size [46]. Deletion of several Hippo pathway components also results in ovarian defects, including decreased follicular development, germ cell loss, follicular cysts and ovarian stromal tumors in *Lats1* mutant mice [47, 48] and reduced fertility and early mortality in *Wwtr1* (*Taz*) mutant mice [49, 50]. A study by Kawamura and colleagues shows convincingly that fragmentation of mouse and human ovaries alters the actin cytoskeleton and stimulates nuclear YAP1 accumulation in somatic cells that is required for increased proliferation and follicular development [51]. More recently, two key studies showed that ablation of YAP1 in granulosa cells impairs proliferation and promotes differentiation [52, 53], but the regulation of Hippo signaling in the COC during ovulation was not examined specifically. In agreement with these previous studies, we present evidence supporting a role for the Hippo signaling pathway in mediating the peri-ovulatory transition of cumulus granulosa cells. The findings indicate that in the absence of ovulatory signals, oocyte-secreted factors suppress Hippo signaling in cumulus cells which leads to activation of YAP1, stimulation of cell proliferation and suppression of differentiation. Ovulatory signals cause phosphorylation and degradation of YAP1 which allows terminal differentiation of cumulus cells.

## Methods

### Animals

Female CD1 mice were bred and raised in the research colony of the investigators. Animals were maintained according to the Guide for the Care and Use of Laboratory Animals (Institute for Learning and Animal Research). All animal use was reviewed and approved by the IACUC committee at The Pennsylvania State University. Mice were weaned at 18 days old and primed with PMSG (5 IU) for 48 h before euthanasia and tissue collection. In some experiments, mice were primed with PMSG for 48 h, followed by hCG (5 IU) for 6 or 24 h before tissue collection.

### In-vitro culture of cumulus-oocyte complexes

Cumulus oocyte complexes (COCs) were collected from mice (18 days old) primed with PMSG for 48 h, as described previously [7]. COCs were randomly allocated to experimental groups and each experiment was repeated several times (*N* = 3–6) with freshly collected COC. Briefly, fresh ovaries were placed in bicarbonate-buffered MEM-α medium (Life Technologies, Grand Island, NY), supplemented with 75 mg/L penicillin G, 50 mg/L streptomycin sulfate, 0.23 mM pyruvate and 2 mg/ml BSA, unless otherwise noted. COCs were released from antral follicles by gentle puncture with 25-gauge needles. In some cases, COCs were oocytectomized (OOX) using a narrow bore glass pipet. For co-culture experiment, the following groups were used: (1) Control: 20 intact COCs cultured for 20 h, followed by harvesting of the cumulus cells; (2) OOX: cumulus cells from 20 COCs cultured for 20 h; (3) Co-culture: Cumulus cells from 20 COCs co-cultured with 40 denuded oocytes (2 oocytes/μl). For determination of the effect of EGF signaling on Hippo transcripts, 20 intact COCs per group were cultured for 0, 4, 8, 12 or 16 h with EGF (10 ng/ml). To determine the effect of YAP1 inhibition with verteporfin (VP) on cumulus cell steroidogenesis, 50 intact COCs per group were cultured in MEM-α medium as indicated above, but supplemented with 5% charcoal stripped serum, either in medium alone (control) or in medium containing 200 nM or 1 μM VP for 16 h. Conditioned media were collected for analysis of progesterone content using a progesterone ELISA kit according to the manufacturer’s instructions (Cayman Chemical, Ann Arbor, MI). The absorbance was read by FLUOstar Omega Microplate Reader at a wavelength of 450 nm.

### In-vitro cell culture (monolayer)

Fresh ovaries from unprimed 18-day old mice were placed in MEM-α medium, mural cell clumps were released from the antral follicles by gentle puncture with syringes and needles. Mural cell clumps were collected and pipetted gently to create a single cell suspension. Approximately 2500 cells per well were plated in medium containing 10% FBS in a 384-well plate (Corning CLS 3985) overnight (25 μl/well). The next day, the medium was replaced with medium containing low serum (0.5% FBS) and cells were co-cultured with denuded oocytes (2 oocytes/μl), or VP (200 nM) or both for 48 h. Cell number was determined using the CellTiter 96 Aqueous One Cell Proliferation Assay (Promega, Madison, WI). Absorbance was measured on a FLUOstar Omega Microplate Reader at 490 nm. To determine YAP1 localization, cumulus cells were stripped off 10–20 COC, pipetted briefly and plated on a chambered glass slide in 100 μl medium for 12 h, followed by washing and culturing alone or with oocytes (4 oocytes/μl) for 24 h). At the end of culture, cells were fixed in 4% paraformaldehyde for 30 min and stained by immunofluorescence using YAP1 (Cell Signaling Technology, 14074) and Goat anti-Rabbit Alexafluor-488 secondary antibody (Thermofisher) using standard methods. Slides were mounted with antifade gold (Invitrogen) with DAPI and imaged on an epifluorescent microscope. Brightness and contrast were adjusted identically in all images.

### Total RNA isolation and real-time PCR

Total RNA was isolated from 20 intact COCs (4 experimental replicates, for EGF and SMAD2/3 inhibitor (SB431542) experiments), cumulus cells from 20 COCs (5 experimental replicates, for oocyte co-culture experiments), using RNeasy Microkit (QIAGEN, Valencia, CA). Quantitect Reverse Transcription Kit (QIAGEN) was used to reverse-transcribe total RNA into cDNA. The quantification of transcripts for the Hippo pathway was normalized to the house-keeping gene *Rpl19*, and gene-specific primers used in real-time PCR are shown in Table 1. The relative fold changes in transcripts were measured using the 2^ddCt^ method as described [54].

**Table 1:** Primer Sequences used for qPCR

Gene Symbol	Forward	Reverse
*Yap1*	ATCCCTGATGATGTACCACTGCCA	CAGGAACGTTCAGTTGCGAAAGCA
*Wwtr1 (Taz)*	CAAGCCTGCATTTCTGTGGCAGAT	TCACTGCCTGAGGTCAGCTTTGTA
*Lats1*	GAATGAGCCCGTGAAAGCAACACA	TTCAGACTTCAGACGCTCCATGCT
*Lats2*	TGAAGAGGGCCAAGATGGACAAGT	AGAGCGTGAGTGTCCAGCTTACAA
*Stk4 (Mst1)*	CAGGGCCTGCATAACATTTGCTGT	TTCCTTGTCTGGCAAAGCCCAAAG
*Stk3 (Mst2)*	AAGAGACCTGATCGCAGAAGCCAT	CATTGTGCCCACGCTTTCTGAACT
*Mob1b*	TTGACAGCAGACTTACCACTCCGT	ACACACACACACACGTCTCCTCAT
*Sav1*	TGGTCCCTGCAAATCCCTACCATA	ACTTCAGCATTCCCTGGTACGTGT
*RPL19*	TTCAAAAACAAGCGCATCCT	CTTTCGTGCTTCCTTGGTCT

All sequences are from 5′ to 3′

### Immunoblotting

Cumulus cells from 30 or 75 COCs or 20 μg of whole ovary lysates from eCG or hCG (6 and 24 h) primed female mice were denatured by boiling for 5 min in Laemmli sample buffer (with 5% 2-Mercaptoethanol), followed by quenching on ice and prepared for immunoblotting as previously described [55]. Proteins were separated on a 4–12% Bis-tris gel (Novex NuPAGE) and transferred to PVDF membrane (0.2 μm). The membranes were blocked in TBST+ 5% BSA for 1 h with shaking at room temperature, followed by incubation with 1:1000 diluted phospho-LATS1 (Ser 909) (Cell Signaling Technology, 9157), phospho-YAP1 (Serine 127) (Cell Signaling Technology, 13008), phospho-TAZ (Ser 89) (Santa Cruz, 17610), YAP1 (Cell Signaling Technology, 14074), TAZ (Abcam, ab84927) or β-actin (ACTB, 1:6000, Sigma) antibodies with agitation at 4 °C overnight. Following incubation, blots were washed 3–4 times, 10 min each with 1 X TBST, and incubated with HRP-labeled secondary antibody (1:50,000) for 1 h at room temperature in the dark. Blots were washed and Pierce ECL Plus substrate (Life Technologies, 80197) was added for 5 min before detecting signal in a phosphorimager (GE STORM 860) or a Bio-Rad XRS+ gel documentation system.

### Statistical analyses

Data were analyzed by either one-way ANOVA followed by Tukey’s post-hoc test, Dunnett’s post-hoc test or student’s t-test as indicated in the figure legends. Minitab 17.1 software and Microsoft excel were used for all analyses. A *p*-value < 0.05 was considered statistically significant.

## Results

### Oocytes suppress Hippo transcripts abundance

**Fig. 1:**
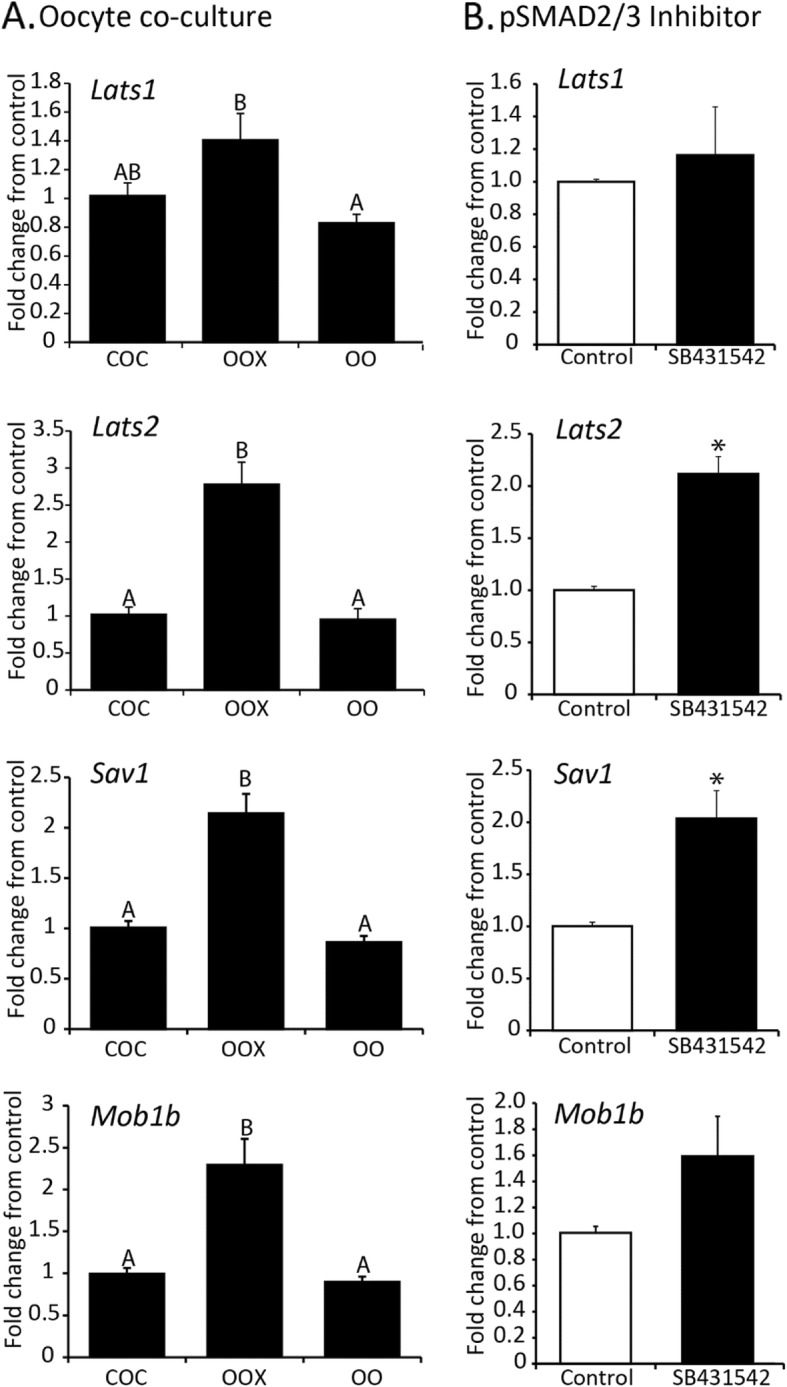
Effect of oocytes and pSMAD2/3 inhibitor on the abundance of Hippo transcripts in cumulus cells **a**. Abundance of *Lats1, Lats2, Sav1 and Mob1b* transcripts in cumulus cells from intact cumulus-oocyte complexes (COC), oocytectomized COC (OOX) and OOX co-cultured with fully-grown oocytes (OO) for 20 h. **b**. Abundance of *Lats1, Lats2, Sav1 and Mob1b* transcripts in COCs cultured alone (control) or with the pSMAD2/3 inhibitor, SB431542 (10 μM) for 16 h. Values are mean ± SEM, *N* = 4–5. ^A,B^ indicates significant differences by one-way ANOVA followed by Tukey’s post-hoc test, *P* < 0.05. *Indicates significant differences from control by Student’s T-test, *P* < 0.05

The relative transcript level (fold change from control) of Hippo transcripts were compared among COC, OOX and OO groups after 20 h of culture. Results showed that compared to COC group, the adaptor genes *Sav1* and *Mob1b* were significantly increased in OOX group, but levels returned to baseline after oocyte co-culture (*P* < 0.05) (Fig. 1a). Similar expression patterns were observed for *Lats1*, and *Lats2* (Fig. 1a). However, expression of *Stk3*, *Yap1* and *Wwtr1* (Taz) mRNA were not significantly different between any of the treatment groups (data not shown). Oocytes activate SMAD2/3 signaling in cumulus cells [7]. To test whether blocking SMAD2/3 signaling with the inhibitor SB431542, increased Hippo transcript abundance, COCs were cultured alone or with SB431542 (10 μM) for 16 h. The adapter gene *Sav1* and upstream kinase *Lats2* were increased approximately two-fold by treatment with the inhibitor, while there was no change in *Mob1b* or *Lats1* (Fig. 1b).

### Verteporfin abrogates the growth promoting effect of oocytes on granulosa cells

**Fig. 2:**
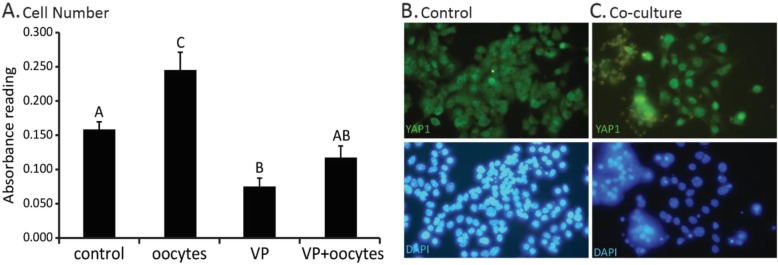
Effect of verteporfin and oocytes on monolayer granulosa cell survival. **a**. Optical density of granulosa cells cultured in medium containing low (0.5%) fetal bovine serum (control) or medium supplemented with oocytes (2 oocytes/μl) and/or verteporfin (200 nM) for 48 h. **b**. YAP1 localization in cumulus granulosa cells cultured alone for 24 h and stained for total YAP1 and DNA (DAPI). **c**. YAP1 localization in cumulus granulosa cells cultured with oocytes (4 oocytes/μl) for 24 h and stained for total YAP1 and DNA (DAPI). Values are mean ± SEM of background subtracted optical density readings. ^A,B,C^ Indicate significant differences by one-way ANOVA followed by Tukey’s post-hoc test, *P* < 0.01, *N* = 3

Verteporfin (VP) acts as a small molecule YAP-TEAD inhibitor, directly inhibiting the binding of YAP1 and TEAD [56]. To examine the effect of VP on oocyte-induced cell proliferation, mural granulosa cells were cultured alone or with oocytes in the presence or absence of VP for 48 h. As expected, VP (200 nM) caused a decrease in cell number (*P* < 0.01) compared to untreated cells whereas oocyte co-culture significantly increased cell number compared to mural cells cultured alone (P < 0.01) (Fig. 2). However, VP (200 nM) completely blocked the ability of oocytes to stimulate cell proliferation (P < 0.01). Consistent with oocyte induced-YAP1 activation, we observed that YAP1 localized to both the nucleus and cytoplasm in granulosa cells cultured alone, but was mainly nuclear in the co-culture group (Fig. 2).

### Verteporfin induces premature differentiation of cumulus cells in vitro

**Fig. 3:**
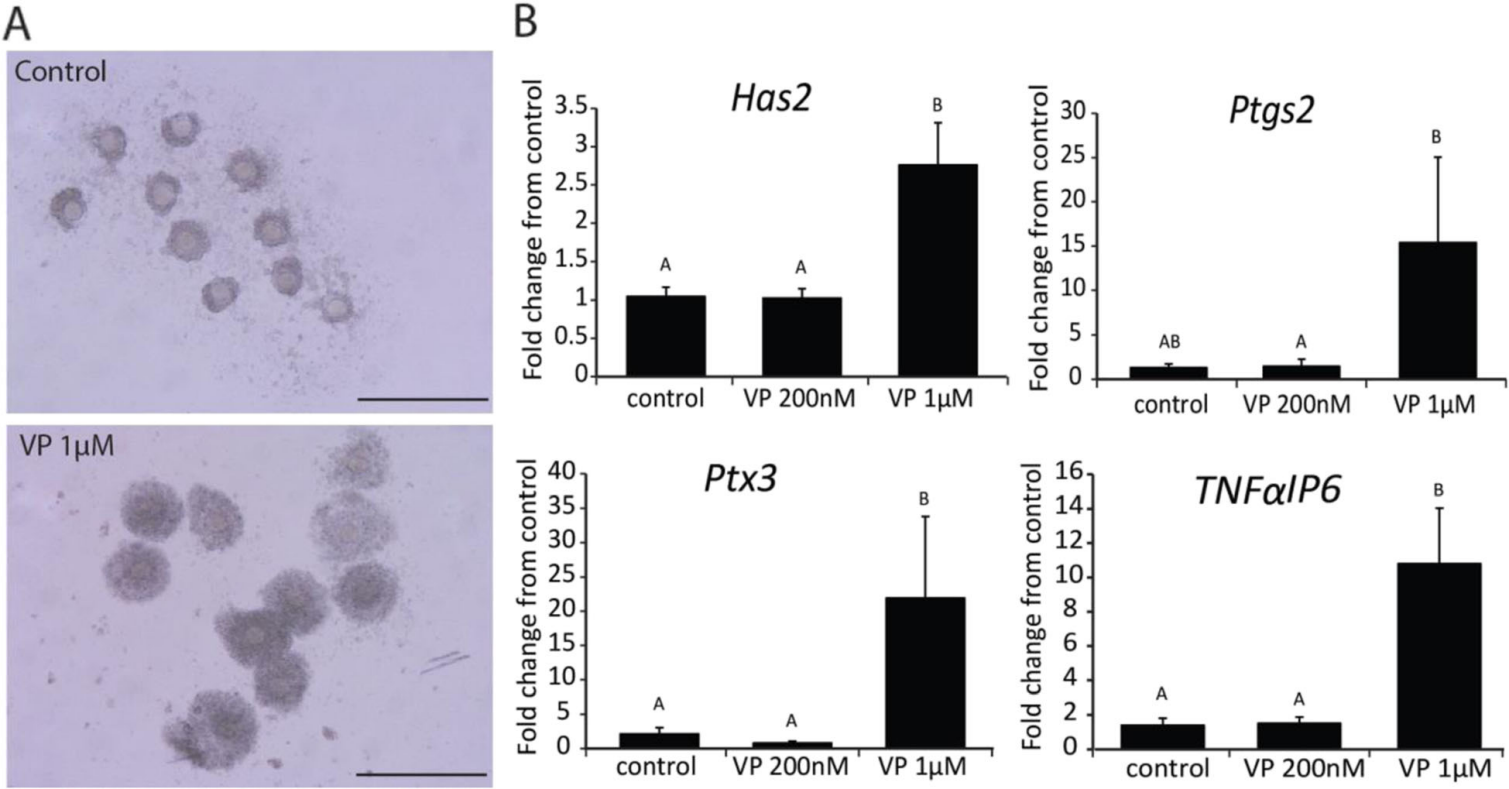
Dosage-dependent effect of verteporfin on the cumulus cell expansion **a**. Representative bright field images of freshly collected COCs treated with control medium or medium containing verteporfin (1 μM) for 16 h, scale = 100 μm. **b**. Fold change of cumulus expansion markers (*Has2, Ptgs2, Ptx3, Tnfaip6*) in COCs treated with verteporfin (200 nM or 1 μM) for 16 h, *N* = 6. ^A,B^ indicates significant differences by one-way ANOVA followed by Tukey’s post-hoc test, P < 0.05

**Fig. 4:**
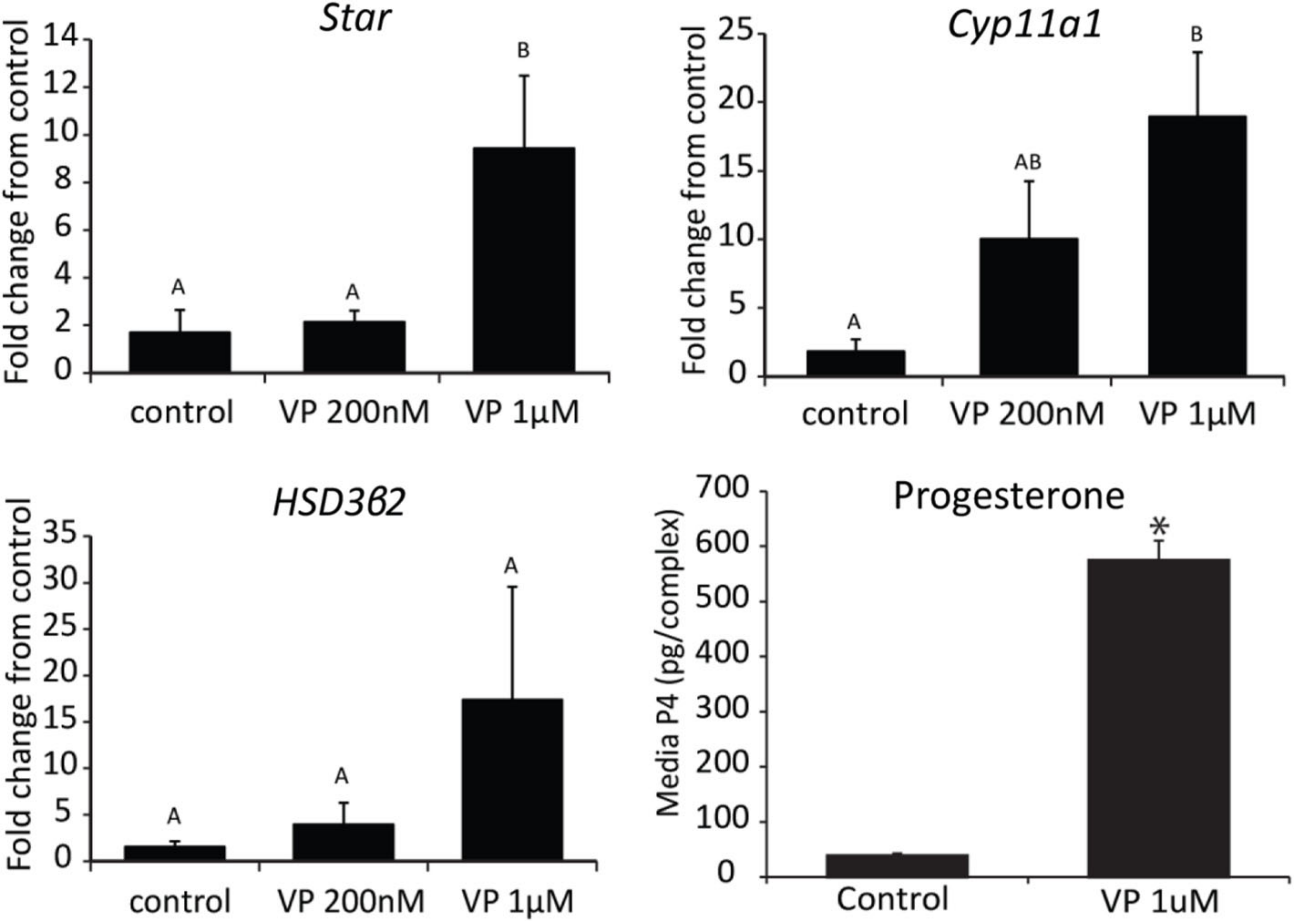
Dosage-dependent effect of verteporfin on the steroidogenesis of COCs. Fold change of steroidogenic transcripts (*Star, Cyp11a1, Hsd3β2*) in COCs treated with verteporfin (200 nM or 1 μM) for 16 h, N = 3–9 and media progesterone (pg/complex) in conditioned media from COC (2 COCs/μl) treated with medium only (control) or verteporfin (1 μM) for 16 h, N = 6. Values are mean ± SEM. ^A,B^ indicates significant differences by one-way ANOVA followed by Tukey’s post-hoc test, P < 0.05. *Indicates significant differences from control by Student’s T-test, P < 0.05

Bright field images (Fig. 3a) showed that cumulus cells treated with VP (1 μM) for 16 h underwent what appeared to be normal cumulus expansion without any ovulatory signals. This phenomenon was absent in control COCs. Consistent with the morphological cumulus expansion caused by VP (1 μM), expansion transcripts (*Has2, Ptgs2, Ptx3, Tnfαip6*) increased 3–20 fold when treated with 1 μM, but not 200 nM VP (Fig. 3b). In addition to cumulus expansion, 1 μM induced a significant increase in key steroidogenic transcripts including *Star* and *Cyp11a1* but not *Hsd3β2* mRNA (Fig. 4). Consistent with an increase in *Star* mRNA, cells treated with 1 μM VP secreted significantly higher progesterone than in the control groups (Fig. 4).

### Ovulatory signals robustly alter the abundance of Hippo transcripts and proteins

**Fig. 5:**
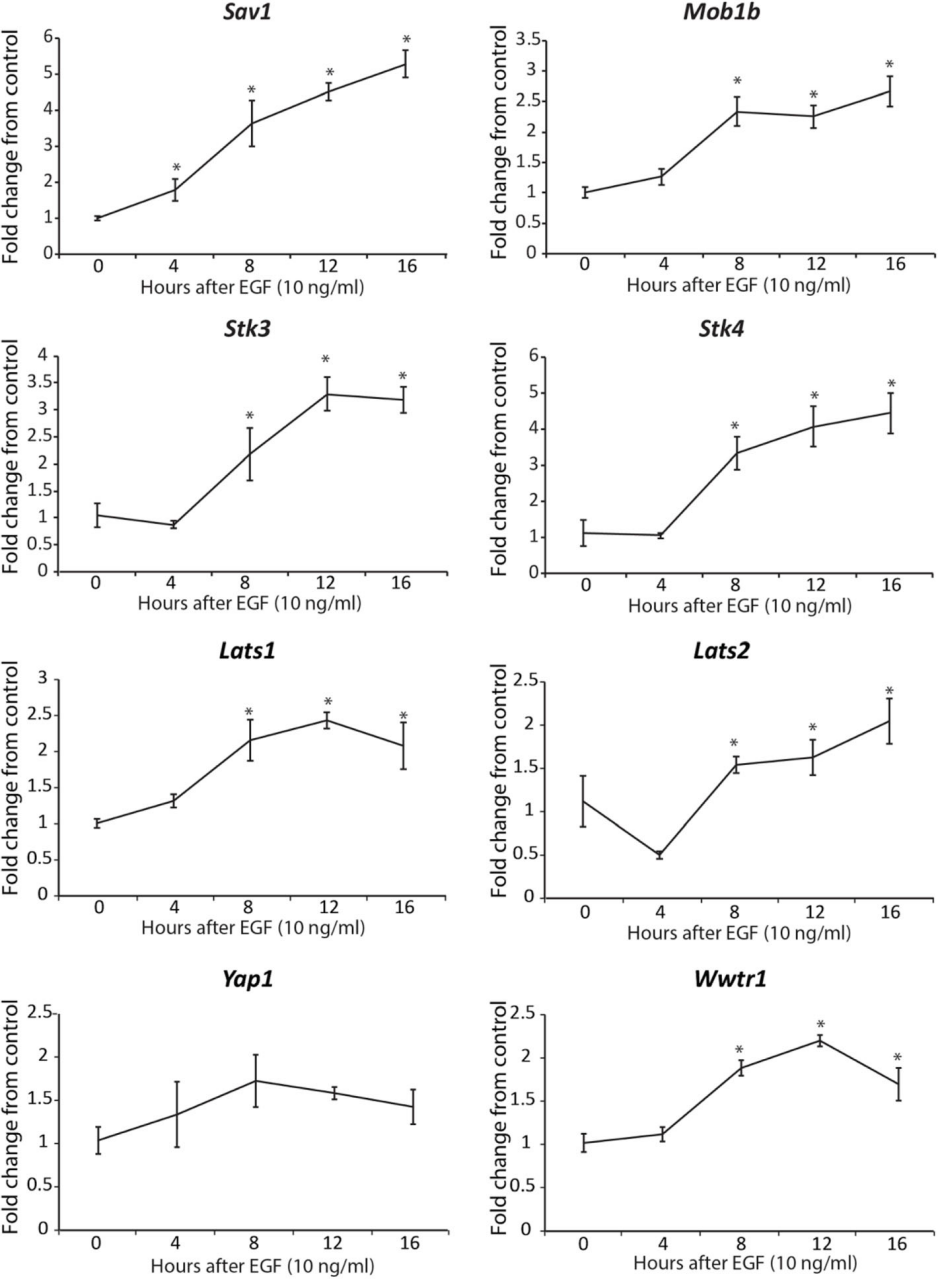
Time-dependent effect of EGF on the abundance of Hippo transcripts in COCs. Abundance of *Sav1, Mob1b, Stk4, Stk3, Lats1, Lats2, Yap1* and *Wwtr1* transcripts in COCs cultured alone (control) or with EGF (10 ng/ml) for 0, 4, 8, 12 or 16 h. Values are mean ± SEM. *Indicates significant differences from control by one-way ANOVA followed by Dunnett’s post-hoc test, P < 0.05, *N* ≥ 3

**Fig. 6:**
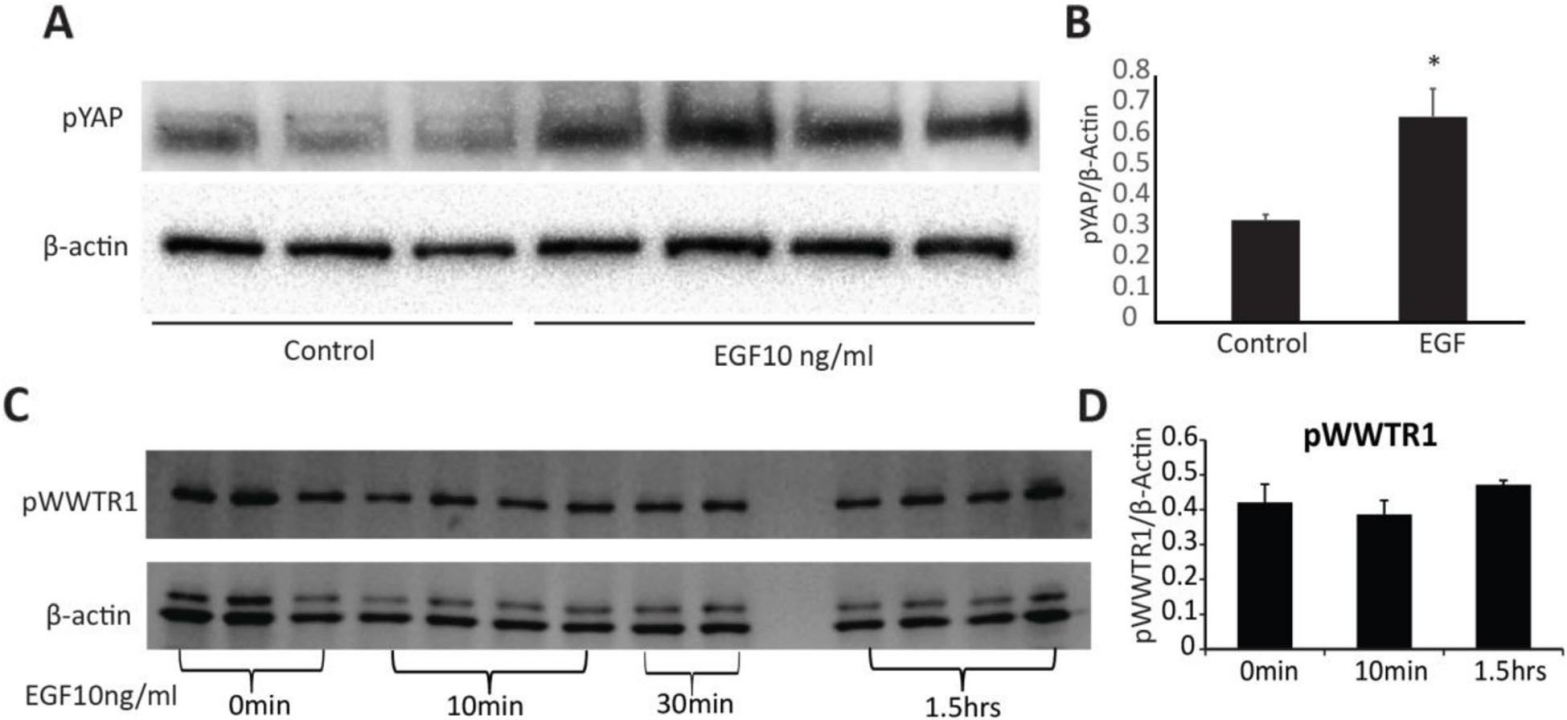
Time-dependent short-term effect of EGF on the expression of Hippo proteins in COCs **a**. Immunoblot showing pYAP (Ser127) and β-actin in COCs treated with EGF (10 ng/ml) for 0 and 60 min. **b**. Densitometric ratio of YAP1 relative to β-actin, N = 3–4. **c**. Immunoblotting showing pWWTR1 (Ser89) and β-actin in COCs treated with EGF (10 ng/ml) for 0, 10, 30 min and 1.5 h. **d**. Densitometric ratio of pWWTR1 relative to β-actin. N = 3–4, 30 min time point was excluded from analysis because there were only 2 observations. Values are mean ± SEM. ^*^ indicate significant differences by student’s t-test, P < 0.05

**Fig. 7:**
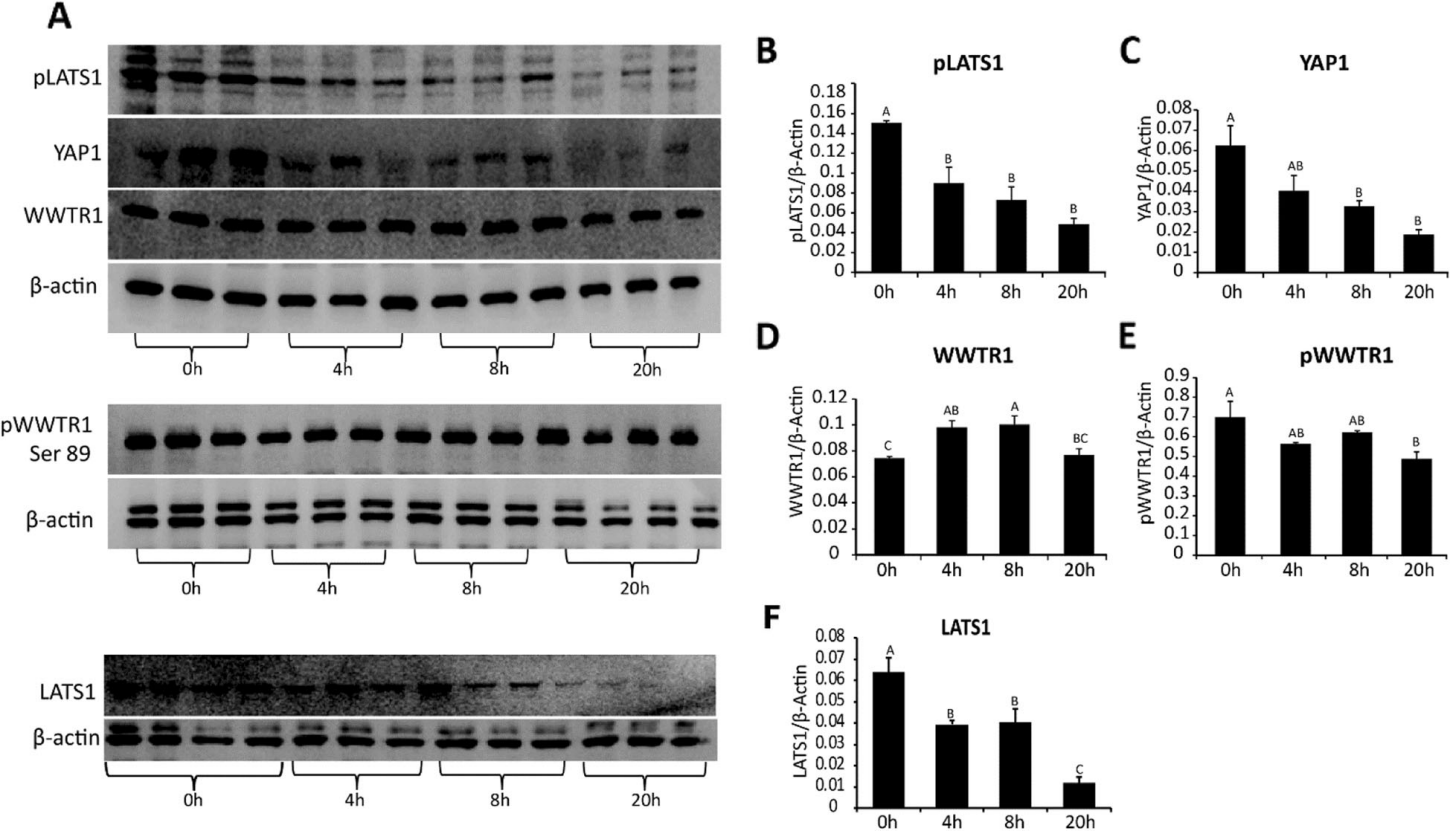
Time-dependent extended effect of EGF on the expression of Hippo proteins in COCs **a**. Immunoblotting for pLATS1 (Ser909), YAP1, WWTR1, LATS1 and β-actin in COCs treated with EGF (10 ng/ml) for 0, 4, 8, 20 h. **b**–**f**. Densitometric band density for pLATS1 (B), YAP1 (C), WWTR1 (D), pWWTR1 (E) and LATS1 (F) normalized to β-actin, N = 3–4. Values are mean ± SEM. ^A,B,C^ indicate significant differences by one-way ANOVA followed by Tukey’s post-hoc test, P < 0.05

**Fig. 8:**
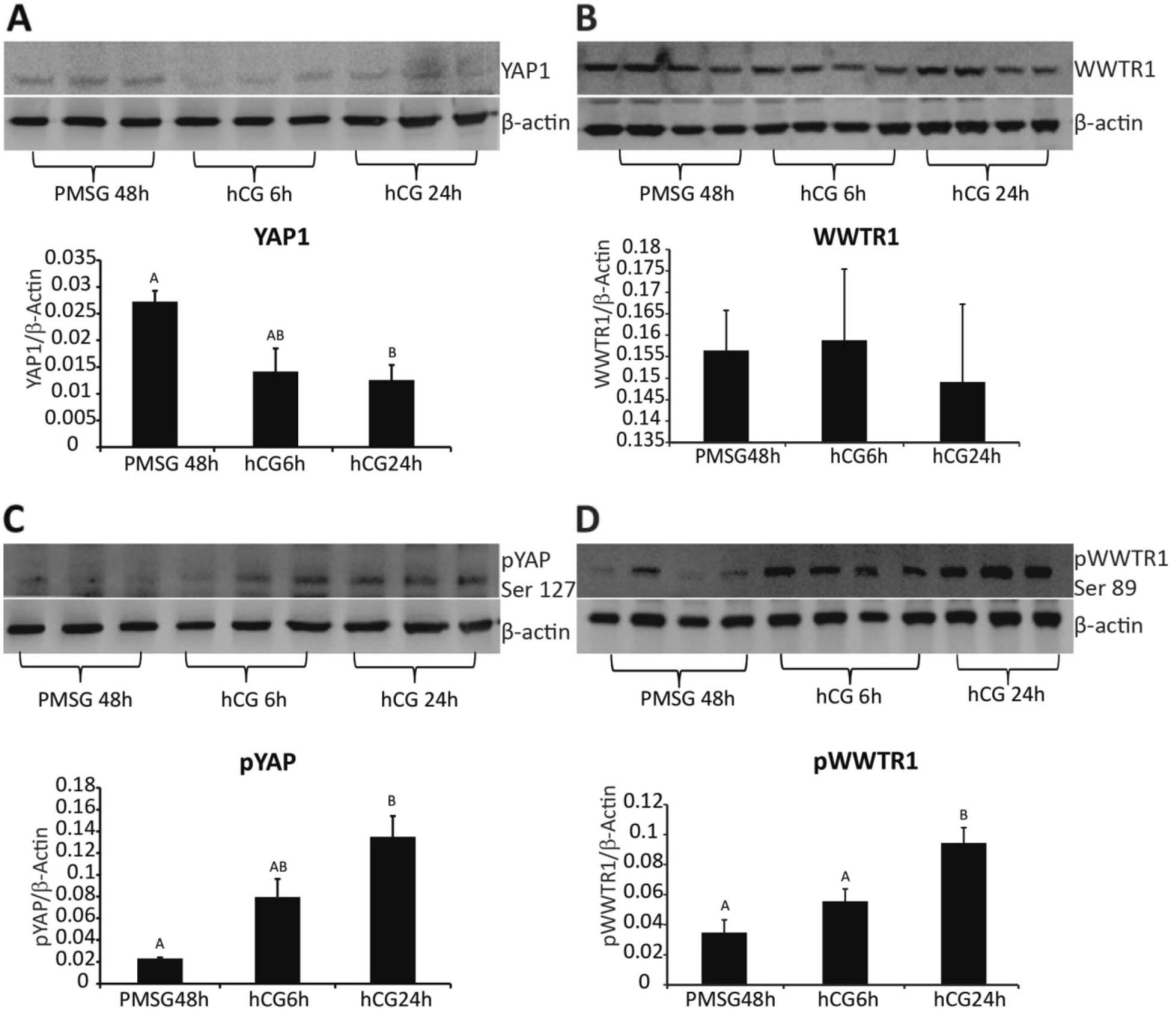
Time-dependent effect of superovulation on the expression of Hippo proteins in COCs Immunoblotting for **a** YAP1, **b** WWTR1, **c** Ser 127 pYAP and **d** Ser 89 pWWTR1 of ovary extracts from mice primed with PMSG for 48 h, or PMSG plus 6 or 24 h after hCG injection. The band density of each sample was normalized to β-actin, N = 3–4. Values are mean ± SEM. ^A,B^ indicate significant differences by one-way ANOVA followed by Tukey’s post-hoc test, P < 0.05

To examine the possible regulation of Hippo transcripts during in vitro maturation, COCs were treated with EGF (10 ng/ml) for 4, 8, 12 and 16 h. The transcript abundance for *Mob1b, Stk3/4, Lats1, Lats2 and Wwtr1* were all significantly increased by 8 h of culture with EGF, while *Sav1* increased by 4 h and *Yap1* was not changed within 16 h after treatment (*P* < 0.05) (Fig. 5). To determine whether acute or sustained EGF signaling affects activation of the Hippo pathway, COCs were treated acutely with EGF for 1 h (Fig. 6) or for 0 h, 4 h, 8 h, and 20 h (Fig. 7). After culture, COCs were lysed and subjected to immunoblotting. Acute EGF treatment caused a significant increase in pYAP after 1 h (Fig. 6a and b), while pWWTR1 did not change up to 1.5 h after treatment (Fig. 6). However, prolonged EGF treatment caused a decrease in both total and phosphorylated LATS1 (pLATS1) by 4 h after treatment which remained low at 8 and 20 h (Fig. 7a and b) but had opposite effects on YAP1 and WWTR1 levels. Prolonged EGF treatment significantly decreased total YAP1 by 8 h and remained low until 20 h (Fig. 7a, c). In contrast, WWTR1 was transiently increased by 4 h, but returned to baseline by 20 h after EGF treatment (Fig. 7a, d), while pWWTR1 was decreased by20 hours after treatment (Fig. 7a, e). To quantify possible changes in Hippo pathway activation in vivo, ovarian extracts from PMSG (48 h) and hCG (6 h, 24 h) injected mice were used for immunoblotting experiments. Expression of total WWTR1 remained unchanged between groups (Fig. 8b), while treatment with hCG for 24 h, but not 6 h, caused a significant decrease in total YAP1 (Fig. 8a). In contrast, both pYAP (Ser 127) and pWWTR1 (Ser 89) were increased after hCG treatment for 24 h (Fig. 8c and d).

## Discussion

The Hippo pathway is a key regulator of the cell fate decision to remain quiescent, divide or undergo apoptosis. Recent reports show that Hippo pathway proteins are expressed in the ovary and regulate follicle activation [57] and subsequent growth of follicles [48, 51, 58]. Deletion of *Lats1* results in germ cell loss and formation of ovarian cysts and stromal tumors [47, 48], while ovarian fragmentation leads to YAP1 upregulation and increased follicular development [51, 59]. Injection of lentivirus shRNA against *Yap1* into the ovarian bursa resulted in a reduction in liter size suggesting an impairment of folliculogenesis [60]. More recently, the disruption of YAP1 in granulosa cells in vitro or in vivo results in severely impaired follicular and granulosa cell development [52, 53]. These findings show clearly that the Hippo pathway is essential for ovarian function. However, the specific role of the Hippo pathway in the COC during ovulation has not been explored in detail. In the present study, we uncovered a role of oocytes and ovulatory signals in modulating expression of Hippo transcripts and Hippo pathway activity and YAP1 activation prior to and during ovulation. Before ovulation occurs, oocytes suppress the Hippo pathway to activate YAP1 and increase granulosa cell survival and/or proliferation while suppressing cellular differentiation. During ovulation, these effects are reversed by ovulatory signals that stimulate Hippo pathway activity to first inhibit YAP1 and then cause YAP1 degradation which allows cells to differentiate.

Granulosa cell development and function are regulated by intra-ovarian signals, including oocyte-secreted products and endocrine signals such as gonadotropins. Oocytes play a central role in promoting the proliferation of granulosa cells throughout follicular development. An elegant study using re-aggregation of isolated oocytes and newborn ovarian somatic cells showed that 14 day-old oocytes dramatically accelerate formation of antral follicles compared to oocytes from newborn ovaries [61]. Clearly oocytes potently influence granulosa cell proliferation [3], survival [4] and differentiation [7, 9, 55]. GDF9 and BMP15 are two oocyte-secreted factors which form hetero- and homodimers that potently activate the SMAD2/3 signaling pathway [10]. However, the underlying mechanism of how oocytes stimulate granulosa cell survival and proliferation has not been completely defined. In this study, we found that oocytes negatively regulate the abundance of Hippo pathway transcripts (*Lats1, Lats2, Mob1* and *Sav1*) in cumulus cells. Moreover, blocking SMAD2/3 phosphorylation in intact COCs increased abundance of Hippo pathway transcripts *Lats2* and *Sav1* suggesting that oocytes regulate the Hippo signaling, at least in part, through a SMAD2/3-mediated pathway. Further work is needed to understand if oocyte suppression of Hippo pathway transcripts is through transcriptional or post transcriptional mechanisms, such as changes in mRNA stability. The suppression of Hippo transcripts could lead to robust activation of the transcriptional co-activators *Yap1* and *Wwtr1*, which in turn could stimulate granulosa cell survival. Indeed, our findings are consistent with this model and show that the YAP1 inhibitor, verteporfin, robustly blocked oocytes from inducing cell survival in a coculture assay. Consistent with stimulation of YAP signaling, oocyte co-culture lead to accumulation YAP1 in the nucleus of cumulus cells. This suggests that oocytes secreted factors facilitate a shift of YAP1 from cytoplasm to the nucleus. However, still to be determined are the molecular steps that link oocyte-secreted factors to YAP1 activation in cumulus cells. One possibility is that YAP1 and/or TAZ proteins interact with SMAD proteins since both SMAD2/3 and SMAD1/5/9 proteins have been shown to physically interact with YAP1 and/or WWTR1 in other tissues [62–65] and oocytes activate both pathways in cumulus cells [7, 9]. Similar to our findings with oocyte-secreted factors, endocrine signals such as steroids and gonadotrophins also stimulate granulosa cell proliferation [53].

In addition to promoting cellular proliferation, oocytes also prevent premature differentiation of cumulus cell. This work was pioneered by Nalbandov and colleagues who demonstrated that oocytes secrete an “anti-luteinization” factor that blocks progesterone production from cumulus cells [5]. Similar effects also occur in other species [66, 67] suggesting this is a conserved mechanism. Recently, YAP1 has been shown to maintain pluripotency of embryonic stem cells [44, 68], and can block differentiation of tissue specific progenitor cells such as myoblasts [69], pancreatic acinar cells [70] and neuronal cells [71–73]. Thus, YAP1 regulates cell fate in various contexts. We tested the effect of the YAP1 inhibitor, verteporfin, on progesterone production and cumulus expansion, two hallmarks of terminal differentiation in cumulus cells. Consistent with a role for YAP1 in blocking cell differentiation, we found that treatment with VP induced premature differentiation of cumulus cells. Remarkably, VP induced expression of expansion-related transcripts and morphological indications of cumulus expansion in the absence of any ovulatory signals. However, the induction of expansion transcripts was not as robust as observed during normal cumulus expansion [7], suggesting that ovulatory signals are also required to fully induce the cumulus expansion response. Treatment with verteporfin also induced the expression of transcripts involved in steroidogenesis and increased progesterone concentration in culture media. Thus, our findings support a model where oocyte stimulated YAP1 activity is required to both promote cellular survival and inhibit premature differentiation of cumulus cells before ovulation occurs. Identification of the oocyte-derived signal(s) that activate YAP1 is an important area of investigation and could identify the anti-luteinizing factor previously described [5].

Recent studies reported that both transcripts and proteins of the Hippo pathway are expressed in the ovary [48, 51, 58]. SAV1, MST1/2, LATS1/2, YAP1 and pYAP proteins are primarily localized to the cytoplasm in granulosa cells, theca cells and oocytes at all stages of follicular development. WWTR1 is strongly nuclear in granulosa cells of follicles of all sizes and in the corpus luteum. In adult human ovaries, YAP1 was recently reported to be primarily nuclear in granulosa cells from primary to pre-ovulatory follicles but it is primarily cytoplasmic in luteal cells [52]. Premature differentiation of COCs treated with VP is consistent with a model that YAP1 blocks cellular differentiation before ovulation. If this model is correct, then it follows that ovulatory signals should upregulate Hippo signaling to terminate YAP1 activity and allow differentiation. To test this idea in vivo, we measured the levels of total and phosphorylated YAP1 and WWTR1 before and after an ovulatory dose of hCG and indeed, consistent with our model, both pYAP and pWWTR1 were increased, while total YAP1 was decreased following an ovulatory dose of hCG. Ovulatory signals regulated the Hippo pathway in a similar way in COCs matured in vitro. In the COCs, EGF increased abundance of several Hippo transcripts 2–5 fold within 16 h. This is consistent with an induction of Hippo pathway activity during ovulation in the COCs and may be due to a decrease in SMAD2/3 activation [7]. However, the increased abundance of Hippo pathway transcripts in the COCs did not translate into an increase in pYAP as we observed in whole ovaries. There was an induction of pYAP at 1 h, indicating greater Hippo signaling activity, but prolonged treatment with EGF led to a decrease in YAP and LATS1 protein in COCs. It is likely that Hippo pathway activity was transiently increased in the COCs to phosphorylate YAP, but this was likely followed by rapid proteosomal degradation of YAP1 protein. Collectively these observations suggest that there are post-translational mechanisms, induced at the time of ovulation, that degrade YAP1 in the follicle to completely silence downstream responses such as proliferation. The proteosomal degradation of YAP1 has been shown in other tissues and cells [74, 75]. Unlike YAP1, levels of total WWTR1 increased slightly after EGF, while pWWTR1 decreased modestly after prolonged EGF treatment. This underscores the potential different roles of YAP1 and WWTR1 in the COCs. In contrast to EGF treatment of COCs in vitro, hCG treatment in vivo caused an increase in both YAP1 and WWTR1 phosphorylation. Nevertheless, in both COCs and the ovary the activity of YAP1 is likely curtailed by either phosphorylation and/or degradation. Overall, these findings fit a working model that predicts YAP1 activity must be abolished at ovulation to allow final cellular differentiation.

**Fig. 9:**
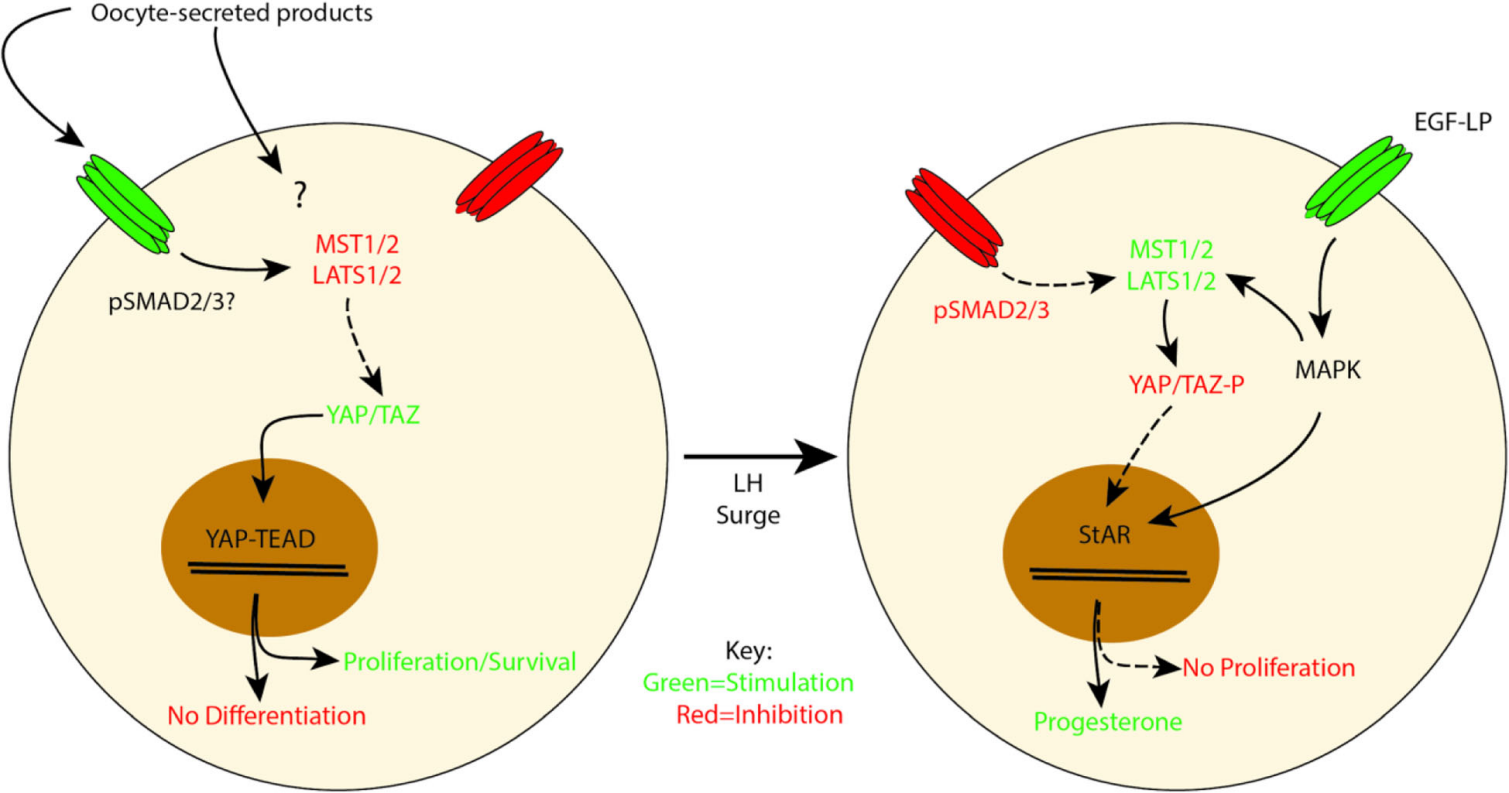
Working model demonstrating the regulation of Hippo signaling during ovulation. Brefore the LH surge, cumulus cells are under the influence of oocyte factors that signal in part through SMAD2/3 mediated pathways to suppress Hippo pathway components and therefore activate YAP1 which stimulates proliferation and/or survival while blocking differentiation. After the LH surge and induction of EGF-like peptides, the Hippo pathway is transiently activated followed by YAP1 degradation which allows cumulus cells to terminally differentiate

Findings from the present study are consistent with a role for the Hippo pathway and the transcriptional co-activator, YAP1, during the periovulatory transition as shown in a working model in Fig. 9. Before ovulation, oocytes prevent premature differentiation and potently stimulate granulosa cell survival through a YAP1-dependent mechanism, while after ovulation YAP-1 is degraded thereby allowing terminal differentiation of follicular cells. Indeed the observation that *Yap1* deletion in luteinizing and luteal cells has no effect on fertility argues that at ovulation YAP1 activity is turned off and no longer required for normal ovarian function [52]. The data with verteporfin should be interpreted with some caution since there is a report of YAP1 independent effects of the drug in cancer cells [76]. Nevertheless, since initially identified [56], verteporfin has been shown to act mainly as a YAP1-TEAD inhibitor in many contexts including the ovary [52, 77, 78]. Moreover, our findings that verteporfin interferes with the growth-promoting effect of oocytes and induces premature differentiation of cumulus cells are consistent with important recent studies showing similar effect in mural granulosa cells in vitro where *Yap1* mRNA is knocked down or in vivo where the *Yap1* gene is knocked out in granulosa cells [52, 53].

## Conclusions

Collectively, the findings from the present study detail a role for oocyte factors and ovulatory signals in modulating the Hippo pathway and YAP1 activation in cumulus granulosa cells before and during ovulation in mice. Specifically, oocytes activate YAP1 signaling thereby promoting granulosa cell survival and proliferation while suppressing cell differentiation. Ovulatory signals inhibit YAP1 activity to allow cells to exit the cell cycle and terminally differentiate. This study mainly focused on the regulatory mechanisms in the COC. However, the present findings together with recent studies showing similar effects after YAP1 ablation in vitro or in vivo [52, 53] and previous studies showing that ovarian fragmentation upregulates YAP1 activity and promotes follicular development [51], demonstrate that the Hippo signaling pathway is a master switch controlling cell fate decisions of granulosa cells during the ovulatory transition.

## Data Availability

Not applicable

## Abbreviations

COC: Cumulus Oocyte Complex
EGF: Epidermal Growth Factor
hCG: human chorionic gonadotropin
PMSG: pregnant mare serum gonadotropin
VP: verteporfin
